# Aromatherapy for Symptom Relief in Patients with Burn: A Systematic Review and Meta-Analysis

**DOI:** 10.3390/medicina58010001

**Published:** 2021-12-21

**Authors:** Hye Won Lee, Lin Ang, Jung Tae Kim, Myeong Soo Lee

**Affiliations:** 1 Herbal Medicine Research Division, Korea Institute of Oriental Medicine, Daejeon 34054, Korea; hwlee@kiom.re.kr; 2 Korean Medicine Science Research Division, Korea Institute of Oriental Medicine, Daejeon 34054, Korea; anglin2808@kiom.re.kr; 3 Korean Convergence Medicine, University of Science and Technology, Daejeon 34113, Korea; 4 The I-MOM Korean Medicine Clinic, Jeju 63232, Korea; ta9989018@hanmail.net; 5 Department of Korean Pediatrics, School of Korean Medicine, Pusan National University, Yangsan 50612, Korea

**Keywords:** anxiety, aromatherapy, burns, pain, systematic review, evidence synthesis

## Abstract

*Background and Objectives*: This review aimed to provide an updated review of evidence regarding the effects of aromatherapy in relieving symptoms of burn injuries, focusing on pain and physiological distress. *Materials and Methods*: Fifteen databases (including five English databases, four Korean medical databases, and four Iranian databases) and trial registries were searched for studies published between their dates of inception and July 2021. Two review authors individually performed study selection, data extraction, and risk of bias assessment, and any discrepancies were solved by a third review author. *Results*: Eight RCTs met our inclusion criteria and were analyzed in this updated systematic review. Our meta-analyses revealed that inhaled aromatherapy plus routine care showed beneficial effects in relieving pain after dressing, as compared to placebo plus routine care (*p* < 0.00001) and routine care alone (*p* = 0.02). Additionally, inhaled aromatherapy plus routine care (*p* < 0.00001) and aromatherapy massage plus routine care (*p* < 0.0001) also showed superior effects in calming anxiety, as compared to routine care alone. None of the included studies reported on AEs. Overall, the risk of bias across the studies was concerning. *Conclusions*: This updated review and synthesis of the studies had brought a more detailed understanding of the potential application of aromatherapy for easing the pain and anxiety of burn patients.

## 1. Introduction

The management of burn injury is a long-term process and requires the same priorities as all other trauma patients. Depending on how deeply and severely a burn penetrates the skin’s surface, burn injury is classified as first-, second-, or third-degree. A first-degree burn usually involves only the epidermis with redness, pain, dryness at the burn site with no blisters. A second-degree burn affects the epidermis and dermis, resulting in a red, blistering, swollen, and painful burn site. A third-degree burn damages the epidermis and dermis, leaving the burn site white or charred and devoid of sensation due to the loss of nerve endings [1]. Despite the advances in therapeutic techniques, burn patients of different severities still suffer from considerable pain and discomfort. Burn injuries often include physical, physiological, and sociological consequences which result in poor quality of life [2]. Thus, essential oils which are known to possess therapeutic and medicinal properties have become an option for the management of burns [3].

Aromatherapy, also known as essential oil therapy, uses plant, flower, or herb extracts to enhance health and wellbeing [4,5]. Aromatic essential oils have been widely studied for first-degree burn injuries due to their ability to relieve pain, reduce scarring, as well as reduce inflammation and antimicrobial activity [6]. A review study has shown that aromatherapy could alleviate pain and reduce anxiety by stimulating the parasympathetic nervous system [7]. Moreover, studies have also been shown that essential oils contain chemical constituents with analgesic-like activity and inhalation of essential oils could stimulate the brain to exert neurotransmitters through olfactory system [8,9]. Aromatherapy also contains oxides that have been found to be analgesic [10]. Essential oils could also stimulate endorphin production, resulting in effects such as pain-reducing, stress releasing, relaxed feeling, and alert enhancement [11]. A systematic review also has shown that essential oils contain bioactive constituents with anxiolytic-like activity [12]. A few studies also reported that aromatherapy alleviate symptoms of cancer such as pain and nausea, and symptoms of behavioral and psychological associated with dementia [13,14,15,16,17,18].

Our previous systematic review has concluded that the evidence of aromatherapy in effectively relieving the symptoms of burn injuries is insufficient [19]. At the time of the original publication, there were only four studies eligible to be included in our final results and we were not able to perform a quantitative synthesis. As publications of clinical studies gradually emerge throughout the years, it justifies the need for an updated systematic review. In this current review, we aimed to provide an updated review of evidence regarding the effects of aromatherapy in relieving symptoms of burn injuries, focusing on pain and physiological distress.

## 2. Methods

The present review is an update of our previously published systematic review and has followed the Preferred Reporting Items for Systematic Reviews and Meta-analyses (PRISMA) reporting guideline [20].

### 2.1. Data Source, Search Strategy, and Study Selection

The following electronic databases were searched from their inception to July 2021: AMED (EBSCO), EMBASE (EBSCO), MEDLINE (PUBMED), The Cochrane Central Register of Controlled Trials (CENTRAL), six Korean medical databases (Korea Med, Oriental Medicine Advanced Searching Integrated System (OASIS), DBPIA, Korean Medical Database (KM base), Research Information Service System (RISS) and the Korean Studies Information Services System (KISS)) and four Iranian databases (Scientific Information Database (SID), IranDoc, MagIran, and IranMedex). For the search strategy, the Medical Subject Headings (MeSHs) “aromatherapy” or “essential oil” AND ‘burns” OR “burns wound” were used. In addition, the reference lists of the potentially eligible articles were searched manually for further relevant reports. No restriction on publication year and languages.

### 2.2. Inclusion and Exclusion Criteria

#### 2.2.1. Design

Only randomized controlled trials (RCTs) were eligible for inclusion and other types of clinical studies were excluded. Conference abstracts, commentaries, letters, dissertations, and thesis were also excluded.

#### 2.2.2. Population

Patients with burn injuries regardless of burn severity, age, gender, and ethnicity were eligible.

#### 2.2.3. Intervention

Any type of aromatherapy regardless of essential oil types, administered route, preparation/processed method, and dosage.

#### 2.2.4. Comparators

Only studies that used placebo and standard/routine care were included. Studies that used other types of aromatherapies as comparators were not eligible.

#### 2.2.5. Outcome Measures

The primary outcomes were pain (measured with validated instruments) and symptoms associated with psychological distress. The secondary outcome of this review was adverse events (AEs)

### 2.3. Study Selection

The titles and abstracts of all the papers identified through the above electronic and manual searches were screened to determine if the studies in question were eligible for inclusion in the review. Potentially relevant articles were subsequently retrieved, and their texts were read in full to determine if they met the abovementioned inclusion criteria. These processes were conducted by two reviewers, and their results were subsequently validated by a third reviewer (MSL). Disagreements between the reviewers regarding study inclusion were resolved through discussion.

### 2.4. Data Extraction

Data extraction was performed by two independent reviewers (HWL and MSL) using a predefined form, and the results of the procedure were subsequently validated by the abovementioned third reviewer. The following information was extracted from each trial included in the review: the first author and year of publication, sample size, mean age, therapeutic regimen, control intervention, primary outcome measures, and main results, as well as the results summary and data regarding AEs. Data regarding changes in symptom severity were also extracted. If such data were reported at different intervals during the treatment periods of the included studies, only the total mean change or the final mean change in symptom severity was used for analysis.

### 2.5. Risk of Bias Assessment

The Cochrane collaboration risk of bias (ROB) assessment tool, a validated research tool used to determine if study results have been affected by selection bias, performance bias, detection bias, attrition bias, reporting bias or another form of bias, was used to assess the ROB in the included studies. The ROB in each of the above domains was scored as high (−), low (+) or unclear (?). The ROB assessments were performed independently by two reviewers (HWL and JK). Any disagreements were resolved through discussion [20].

### 2.6. Data Synthesis

The RCTs were clinically heterogeneous with respect to the type of interventions (plant oil), controls and outcomes used therein. Therefore, we decided to perform a qualitative review rather than pool the data statistically. The estimated effect sizes for each treatment and control intervention evaluated in the included studies were calculated and compared using Review Manager 5.1 (Copenhagen: The Nordic Cochrane Center, Cochrane Collaboration, 2011) [20].

## 3. Results

### 3.1. Description of Included SRs

**Figure 1:**
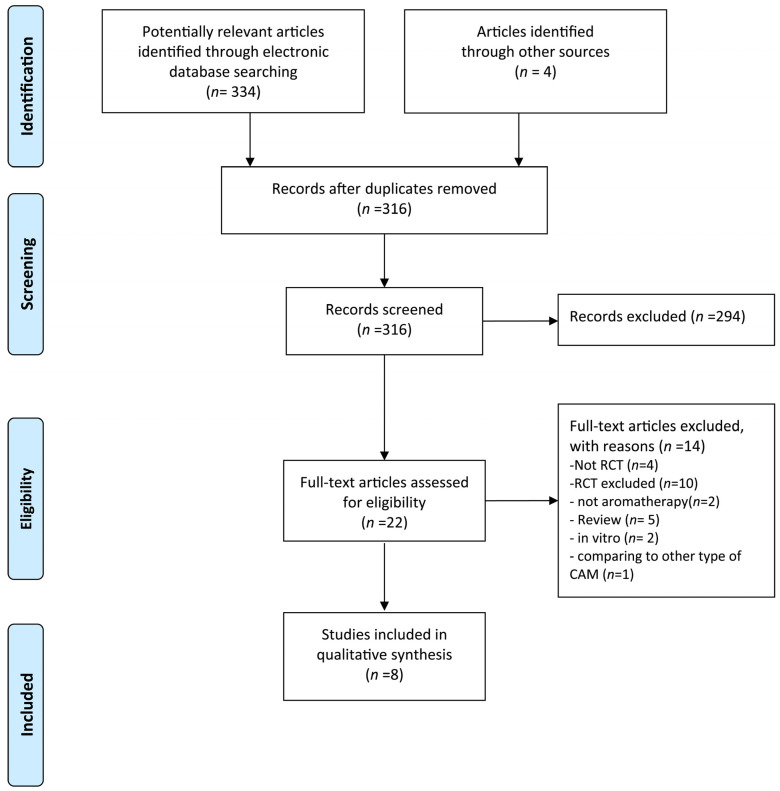
PRISMA diagram for the included studies. CAM: complementary and alternative medicine; RCT: randomized controlled trial.

Our initial search yielded 338 records and 316 records were screened after removing duplicates. A total of eight studies were finally included in this updated review [21,22,23,24,25,26,27,28] (Figure 1). Table 1 summarized the key data pertaining to the included studies. All the RCTs included in this study were conducted in Iran. Five studies used three-armed [22,23,26,27,28], two studies used two-armed [21,25], and one study used four-armed parallel design [24]. For interventions, two studies [23,25] used lavender oil, three studies used (damask) rose oil [21,22,24], and three studies [26,27,28] used a mixture of several essential oils. Aromatherapy was administered via inhalation for five studies [21,22,23,24,25], massage for two studies, [27,28] and both inhalation and massage for one study [26]. Controls included placebo, routine care, and standard nursing care. Besides, all the RCTs registered in trial registries where six trials [21,22,23,24,25,26] registered while recruiting and two trials [27,28] registered prospectively. The summary of ongoing studies relevant to this topic was described in Table 2.

### 3.2. Risk of Bias Assessment

**Figure 2:**
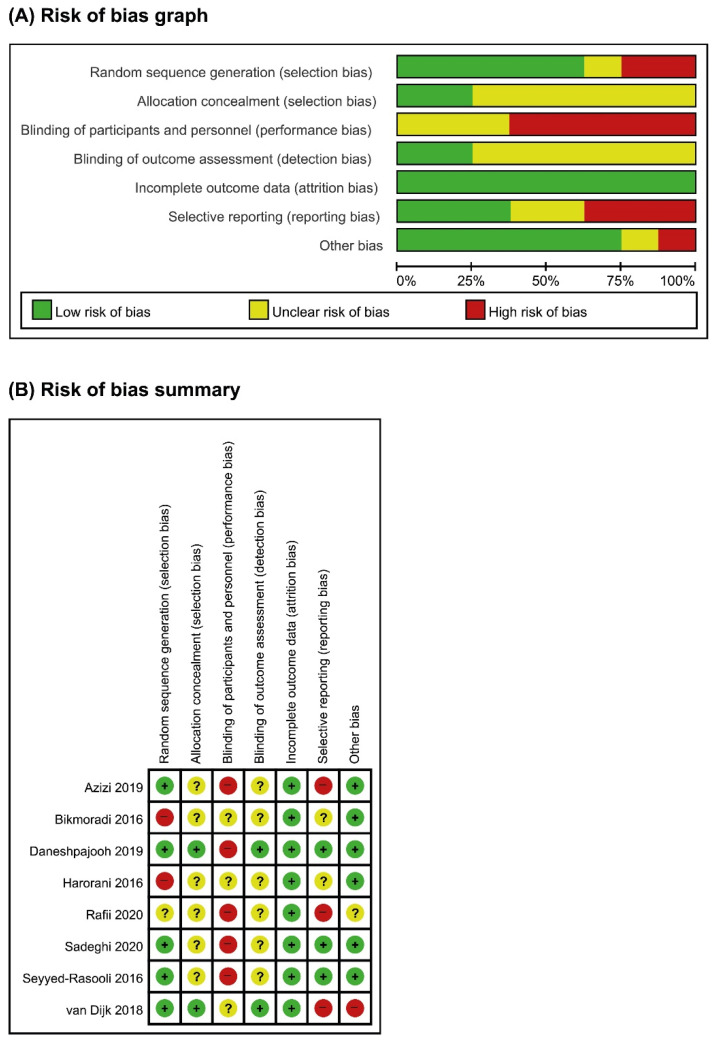
(**A**) Risk of bias graph: review authors’ judgments about each item’s risk of bias item presented as percentage across all included studies; (**B**) risk of bias summary: review authors’ judgments about each item’s risk of bias for each included study (+, low risk; ?, unclear; −, high risk).

For random sequence generation, five studies [22,23,24,26,28] were assessed as low risk of bias, one study [27] as unclear, and the remaining two studies as high risk [21,25] (Figure 2). Most trials did not report on allocation concealment and were judged as unclear risk of bias, except for two trials [24,28] which was assessed as low risk. In terms of blinding, five trials [22,23,24,26,27] were assessed as high risk of bias and three studies [21,25,28] as unclear for blinding of participants and personnel whereas most of the trials were accessed as unclear risk of bias for blinding of outcome assessor. For attrition bias, most trials were assessed as low risk of bias except for one [27]. Three trials [22,24,26] were judged as low risk of bias in selective reporting while two trials [21,25] were unclear and three trials [23,27,28] were high risk due to missing outcomes. For other biases, most trials were assessed as low risk except for one [24] where their outcome measurement was unclear. Overall, the methodology of the included trials was less ideal with concerning flaws.

### 3.3. Outcome Measures

#### 3.3.1. Pain

##### Inhaled Aromatherapy plus Routine Care vs. Placebo plus Routine Care

**Figure 3:**
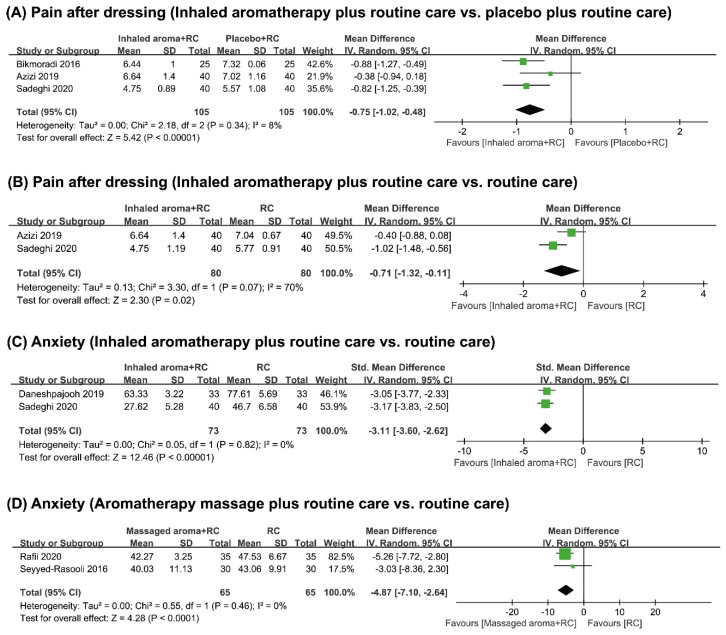
Forrest plot of (**A**) pain outcome after dressing in inhaled aromatherapy plus routine care vs. placebo plus routine care, (**B**) pain outcome after dressing in inhaled aromatherapy plus routine care vs. routine care alone, (**C**) anxiety in inhaled aromatherapy plus routine care vs. routine care alone (after dressing), (**D**) anxiety in aromatherapy massage plus routine care vs. routine care alone.

Three trials tested inhaled aromatherapy plus routine care for pain compared to placebo plus routine care [21,22,23]. All trials showed that inhaled aromatherapy plus routine care significantly reduced pain after dressing and the results of meta-analysis also showed the same results (*n* = 210, mean difference (MD) −0.75, 95% confidence interval (CI) −1.02 to −0.48, *p* < 0.00001, I^2^ = 8%, Figure 3A).

##### Inhaled Aromatherapy plus Routine Care vs. Routine Care Alone

Three trials compared the effects of inhaled aromatherapy plus routine care on reducing pain compared to routine care alone [22,23,26]. Two trials [22,23] measured the pain intensity after dressing while one study [26] measured the general pain intensity. All studies showed significant effects of inhaled aromatherapy plus routine care for pain reduction. The meta-analysis also showed significant pain reduction after dressing by inhaled aromatherapy plus routine care (*n* = 160, MD −0.71, 95% CI −1.32 to −0.11, *p* = 0.02, I^2^ = 70%, Figure 3B).

##### Aromatherapy Massage plus Routine Care vs. Routine Care Alone

One trial [26] compared the effects of aromatherapy massage plus routine care for pain reduction compared with routine care alone and showed a significant difference between the two groups (*n* = 60, MD −2.46 95% CI −3.64 to −1.28, *p* < 0.0001)

#### 3.3.2. Anxiety

##### Inhaled Aromatherapy plus Routine Care vs. Placebo plus Routine Care

Two studies [22,25] tested inhaled aromatherapy plus routine care for anxiety compared to placebo plus routine care. One trial [22] measured anxiety outcome after dressing and one trial [25] measured the general anxiety. Both trials showed inhaled aromatherapy plus routine care significantly reduced anxiety in burn patients after dressing (*n* = 80, MD −15.85, 95% CI −18.35 to −13.35, *p* < 0.00001) and in general (*n* = 60, MD −4.60, 95% CI −7.07 to −2.13, *p* = 0.0003).

##### Inhaled Aromatherapy plus Routine Care vs. Routine Care Alone

Three trials [22,24,26] investigated inhaled aromatherapy plus routine for anxiety, as compared to routine care alone. Two trials [22,24] measured anxiety after dressing and one trial [26] measured anxiety in general. The pooled results showed that inhaled aromatherapy plus routine care significantly reduced anxiety after dressing (*n* = 146, standard mean difference (SMD) −3.11, 95% CI −3.60 to −2.62, *p* < 0.00001, I^2^ = 0%, Figure 3C).

##### Aromatherapy Massage plus Routine Care vs. Placebo plus Routine Care

Only one trial [27] compared the effects of aromatherapy massage plus routine care for general anxiety compared with placebo plus routine care and failed to show a significant difference between the two groups.

##### Aromatherapy Massage plus Routine Care vs. Routine Care Alone

Two trials [26,27] compared the effects of aromatherapy massage plus routine care for general anxiety compared with routine care alone and meta-analysis showed superior effects (*n* = 130, MD −4.87, 95% CI −7.10 to −2.64, *p* < 0.0001, I^2^ = 0%, Figure 3D).

#### 3.3.3. Sleep Quality

##### Aromatherapy Massage plus Routine Care vs. Placebo plus Routine Care

Only one trial [27] investigated the sleep quality between aromatherapy massage plus routine care and placebo plus routine care and showed significant results between two groups (*n* = 70, MD −1.58, 95% CI −2.92 to −0.24, *p* = 0.02).

##### Aromatherapy Massage plus Routine Care vs. Routine Care Alone

One trial [27] compared the effects of aromatherapy massage plus routine care for sleep quality compared with routine care alone also showed significant results between two groups (*n* = 70, MD −1.83, 95% CI −3.30 to −0.36, *p* = 0.01).

#### 3.3.4. Adverse Events

None of the included studies assessed the incidence of AEs.

## 4. Discussion

### 4.1. Summary of the Main Results

Eight studies were included and analyzed in the updated systematic review. Our findings indicate that aromatherapy has the potential in reducing pain and anxiety. Inhaled aromatherapy plus routine care showed beneficial effects in relieving pain after dressing, as compared to placebo plus routine care or routine care alone. Compared to routine care alone, inhaled aromatherapy plus routine care showed superior effects in calming anxiety after dressing. Similarly, aromatherapy massage plus routine care compared with routine care alone also presented favorable effects in easing general anxiety. However, none of the studies reported on AEs and the overall risk of bias across the studies were concerning.

### 4.2. Overall Completeness and Applicability of Evidence

The inclusion of five additional studies has brought a greater understanding of the effects of aromatherapy on burn patients. The inclusion of new studies has enabled us to conduct meta-analyses on two main outcomes of pain and anxiety and revealed the potential role of aromatherapy in alleviating burn symptoms. Yet, most of the included trials have methodological limitations such as small sample sizes, lack of proper reporting, and concerning risk of bias. The aromatherapy intervention used in the included studies also varied in the type of essential oil, dosage, and administration method, limiting our ability to perform further analysis. Although most studies used placebo and/or routine care as the comparator, detailed information relevant to blinding methods is not provided. Successful blinding of participants is considered as a key aspect in methodology assessment of the trials as the smell of essential oils is highly distinguishable. The intervention period is also relatively short which limits us to provide any treatment recommendations.

### 4.3. Agreement or Disagreement with Other Studies or Reviews

In comparison to prior systematic reviews [19], we identified five new RCTs [22,23,24,27,28] and successfully updated the evidence for aromatherapy on this condition. A systematic review [29] studied on the effectiveness of non-pharmacological interventions for anxiety in burn patients reported that aromatherapy presented no evidence of significant effect in reducing anxiety; however, their meta-analysis included all three aromatherapy studies regardless of their administration method and high heterogeneity was found across the trials. In our review, we included eight aromatherapy studies and performed meta-analysis based on different administration methods, and found that inhaled aromatherapy and aromatherapy massage present positive effects in easing anxiety. A recent systematic review [30] investigated the effect of aromatherapy on reducing different types of acute pain concluded that aromatherapy had a favorable effect on alleviating the severity of acute pain. Our findings also showed that aromatherapy had beneficial results in easing pain for burn injuries.

### 4.4. Limitations of This Review

Despite the addition of recent studies, the small sample size and poor methodology of the included studies remain the major limitations of this updated review. The findings of this review should also be interpreted with caution as they may be affected by location and publication bias, as well as the variations in treatment and comparator regimens. The external and internal validity of the trials should also be taken into consideration due to the lack of complete and detailed trial reporting. Moreover, only a few studies were included for meta-analyses which relatively lead to the lack of conclusive findings.

### 4.5. Implications for Practice and Research

The management of burn patients requires delicate care especially in the aspect of pain and anxiety. Although the meta-analyses in this review could only be conducted on limited studies, aromatherapy has shown encouraging effects in easing pain and anxiety and could play a role in complementing daily routine care. In the meantime, more studies are needed to guide the recommendation of aromatherapy in clinical practice. Current available studies are insufficient to demonstrate great significant effects of aromatherapy in managing burn. As management of burn is often long-termed, it is necessary to extend the period of aromatherapy intervention to fully evaluate its effectiveness. Besides, future studies should also consider a single type of essential oil as the smell and function of each type of essential oil are distinct. In general, well-designed RCT with proper reporting, longer intervention period with specific essential oil, and larger sample size are needed to validate the usage and effectiveness of aromatherapy for the management of burn.

## 5. Conclusions

This updated review and synthesis of the studies had brought a more detailed understanding of the potential application of aromatherapy for easing the pain and anxiety of burn patients. Regardless of limited evidence, our findings showed significant improvements in the management of pain and anxiety upon the usage of aromatherapy in burn patients. Studies in compliance with proper guidelines are required to ensure conclusive results and to determine whether aromatherapy is a sustainable option for burn management.

## Figures and Tables

**Table 1:** Summary of randomized clinical studies of aromatherapy for managing symptoms in patients with burns.

First Author (Year) [Ref.]	Total Sample Age (Range or Mean)/ Diagnostic Criteria	Intervention (Regimen)	Control (Regimen)	Main Outcomes	Main Results (Effect Estimate)	Trial Registration Number Registration Time
Bikmoradi (2016) [21]	50 (A: 33, B: 34)/ second degree burn or second and third degree burns	(A) Damask Rose (inhale, 40%, 5 drops, 20 min before dressing, 2 nights, *n* = 25), plus routine care	(B) Placebo (distilled water, 5–30 min, *n* = 25), plus routine care	Pain (VAS) [dressing]	MD-0.88 [−1.27, −0.49], *p* < 0.001	IRCT 201302249759N4 While recruiting
Sadeghi (2020) [22]	120 (A:37, B:37, C:35)/ second degree burns <30%	(A) Damask Rose (inhale, 40%, 6 drops, breath 5 times, 60 min, *n* = 40), plus C	(B) Placebo (distilled water, 6 drops, breath 5 times, 1 h, *n* = 40), plus C (C) Routine care (*n* = 40)	(1) Pain (VAS) (2) Anxiety (STAI) [dressing]	(1) A vs. B, MD-0.82 [−1.25, −0.39], *p* < 0.001; A vs. C, MD-1.02 [−1.48, −0.56], *p* < 0.0001 (2) A vs. B, MD-15.85 [−18.35, −13.35], *p* < 0.00001; A vs. C, MD-3.17 [−3.83,−2.5], *p* < 0.00001	IRCT 2017030632129N3 While recruiting
Azizi (2019) [23]	120 (A:39, B:37, C:34)/ second degree burns <30%	(A) Lavender (inhale, 2%, 10 drops, breath 5 times, 60 min, 1 time, *n* = 40), plus C	(B) Placebo (distilled water, 10 drops, 1 h, 1 time, *n* = 40), plus C (C) Routine care	Pain (VAS) [dressing]	A vs. B, MD-0.38 [−0.94, 0.18], NS; A vs. C, MD-0.40 [−0.88, 0.08], NS	IRCT 2017030632129N3 While recruiting
Daneshpajooh (2019) [24]	140 (A:44, B: 40, C: 41, D: 44)/ second or higher degree burn injury	(A) Rose (inhale, 40%, 5 drops, for 20min, once daily for 3 days, *n* = 33), plus B	(B) Routine care (*n* = 33) (C) Benson relaxation (*n* = 33), plus B (D) A + C (*n* = 33), plus B	Burn specific pain anxiety scale [dressing]	A vs. B, MD-3.05 [−3.77, −2.33], *p* < 0.00001	IRCT 20171212037843N1 While recruiting
Harorani (2016) [25]	60 (18–65)/ second degree burns or second and third degree burns together	(A) Lavender (inhale, 2%, 2 drops, 20 min, 3 days, *n* = 30), plus routine care	(B) Placebo (distilled water, n.r., *n* = 30), plus routine care	Anxiety (STAI) [general]	MD-4.60 [-7.07, -2.13], *p* < 0.001	IRCT 2013042413110N1 While recruiting
Seyyed-Rasooli (2016) [26]	90 (A: 35, B: 35, C: 38)/ second degree burns <20%/	(A) Essential oils (inhale, lavender oil 7 drops and Rosa damascene 3 drops, 30min, n.r., *n* = 30), plus C (B) Essential oils (massage, lavender oil 7 drops and Rosa damascene 3 drops, 30 min, n.r., *n* = 30), plus C	(C) Routine care (*n* = 30)	(1) Pain (VAS) (2) Anxiety (STAI) [general]	(1) A vs. C: MD-1.73 [−3.03, −0.43], *p* < 0.05; B vs. C: −2.46 [−3.64, −1.28], *p* < 0.0001; A vs. B: 0.73 [−0.54, 2.00], NS (2) A vs. C: MD-4.76 [−9.93, 0.41], NS; B vs. C: MD-3.03 [−8.36, 2.30], NS; A vs. B: −1.73 [−7.21, 3.75], NS	IRCT 201404176918N17 While recruiting
Rafii (2020) [27]	105 (A: 36, B: 37, C: 40)/ second- and third-degree burns	(A) Aroma (massage, lavender oil 2 drops and chamomile 2 drops, 20 min, 3 session within 1 week, *n* = 35), plus C	(B) Placebo (massage, bady oil, 20 min, 3 session within 1 week, *n* = 35), plus C (C) Routine care (*n* = 35)	(1) Anxiety (STAI) (2) Sleep [general]	(1) A vs. B: MD-0.82 [−3.30, 1.66], NS; A vs. C: MD-5.26 [−7.72, −2.80], *p* < 0.0001 (2) A vs. B: MD-1.58 [−2.92, −0.24], *p* < 0.05; A vs. C: MD-1.83 [−3.30, −0.36], *p* = 0.01	IRCT 20180120038444N1 Prospective
van Dijk (2018) [28]	287 children (A: 24, B: 28, C: 25 months)/ second- and third-degree burns Burn incident <1 week	(A) Aroma (massage, 1% essential oils (chamomile, lavender, neroli), 10–20 min, 1–5 session within 2 weeks, *n* = 108), plus C	(B) Placebo (massage, carrier oil, 10–20 min, 5 session within 2 weeks, *n* = 90), plus C (C) Standard nursing care (*n* = 86)	(1) MTI, BSC (2) COMFORT-B (3) Distress (NRS) [general]	(1) NA ^†^ (2) NS (3) NS	Trial NL3771 (NTR3929) Prospective

BSC, behavioural relaxation scale; COMFORT-B, COMFORT behavior scale; IRCT, Iranian Registry of Clinical Trials; MTI, muscle tension inventory; n.r., not reported; NA, not available; NRS, numeric rating scale; NS, not significant; STAI, Spielberger state trait anxiety inventory; VAS, visual analog scale; ^†^, not computable due to large missing values.

**Table 2:** Summary of ongoing or not published randomized clinical studies of aromatherapy for patients with burns.

Principal researcher (Year)	Total Sample Age (Range or Mean)/Diagnostic Criteria	Intervention (Regimen)	Control (Regimen)	Main Outcomes	Trial Registration Number
Arjomandzadegan (2017)	60/(14–50 yrs) Second degree burn	(A) Thyme (spray, 1 to 5 times/day, *n* = 30), plus B	(B) Standard care (rinse with saline and silver ointment, *n* = 30)	Grid and depth of wound	IRCT2017032726394N3
Arjomandzadegan (2018)	100/(2–10 yrs) Second degree burns	(A) Thyme (spray, 1 to 2 times/day, *n* = 50), plus rinse with saline and silver ointment	(B) Placebo (distilled gas1 to 2 times/day, *n* = 50), plus rinse with saline and silver ointment	Grid and depth of wound	IRCT20161017030336N1
Froutan (2018)	60/(18–60 yrs) Second and third-degree burns	(A) Essential oils (inhale, Damask Rose (40%, 5 drops), lavender (10%, 7 drops), *n* = 30), plus B	(B) Anesthetic drugs (midazolam, fentanyl and ketamine, *n* = 30)	(1) Reducing anesthetic drugs (2) Brain activity (BIS)	IRCT20171123037599N2

BIS, Bispectral index; IRCT, Iranian Registry of Clinical Trials.

## Data Availability

Data sharing not applicable.
